# A Case of Aripiprazole-Induced Tardive Dyskinesia with Dramatic Evolution

**DOI:** 10.1155/2016/7031245

**Published:** 2016-10-12

**Authors:** Edwige Heitzmann, Hervé Javelot, Luisa Weiner, Bruno Michel

**Affiliations:** ^1^North Alsace Mental Health Clinic (Etablissement Public de Santé Alsace Nord (EPSAN)), Brumath, France; ^2^Psychiatry Department, University Hospital of Strasbourg, Strasbourg, France; ^3^OMEDIT Alsace, Faculté de Pharmacie-Laboratoire HuManiS (EA 7308), Service Pharmacie-CHRU de Strasbourg, Strasbourg, France

## Abstract

Aripiprazole is reported to be a good clinical safety profile antipsychotic. However, recent data suggest that the risk of tardive dyskinesia could be higher than initially thought. We report the case of aripiprazole-induced tardive dyskinesia with dramatic evolution in a patient with several risk factors, including older age and exposure to antipsychotic over a period longer than six months. This case and its dramatic evolution, associated with other cases recently published, suggest reconsidering the real risk of tardive dyskinesia associated with aripiprazole, particularly in the elderly.

## 1. Introduction

Second-generation antipsychotics (SGAs) differ from classical neuroleptics by (i) 5HT_2A_ receptors antagonism and (ii) weaker link capacities and faster dissociation of D_2_ receptors [1]. The specific pharmacology of aripiprazole (5HT_2A_ receptor antagonism and partial agonism including 5HT_1A_ and D_2_ receptors) may justify its classification as a third-generation antipsychotic acting as a “dopaminergic stabilizer” (dopamine agonist or antagonist in hypodopaminergic or hyperdopaminergic states, resp.) [1]. Due to their pharmacological characteristics, SGAs, and more specifically aripiprazole, are associated with a lower theoretical risk of tardive dyskinesia compared to classical neuroleptics. Several publications have even presented clinical cases with an improvement in dyskinesia or dystonia after switching to aripiprazole [2–4]. However, data published since the commercialization of aripiprazole suggest that the risk of tardive dyskinesia could be higher than was initially imagined [1, 5]. We report below a case of aripiprazole-induced tardive dyskinesia with a dramatic evolution following 3 years after treatment interruption.

## 2. Case Description

In 2011, Mr. X, aged 74, with no psychiatric history, consulted a psychiatrist at private practice for a depressive episode with abulia, anhedonia, apathy, and sadness. There was no significant family psychiatric history. Physical symptoms had appeared a few months before as he complained of feeling weak due to chronic anemia secondary to an essential thrombocytosis (JAK2 mutation positive) that showed very stable evolution until the end of his life under hydroxycarbamide. The psychiatric diagnosis made by that time was a severe depressive syndrome associated with major anxiety. During autumn 2011, the patient received a combination of escitalopram 10 mg/d and aripiprazole 5 mg/d. This prescription seemed inappropriate however because there were no delirious or melancholic symptoms, and there was no need for immediate antidepressant potentiation.

In the middle of 2012, nine months after its introduction, aripiprazole was discontinued by his new psychiatrist because of the reasons listed above, the age of the patient, and early abnormal movement with lingual-facial-buccal dyskinesia and choreic movements (lower limb). Depressive symptomatology was partially improved with escitalopram monotherapy 20 mg/d and led to the disappearance of suicidal thoughts, but abulia and anhedonia remained. MRI data revealed no lesion of the basal ganglia and brain stem in particular. In March 2013, despite aripiprazole discontinuation, abnormal movements remained present and led to the introduction of tetrabenazine 37.5 mg/d, associated later with clonazepam 0.6 mg/d and baclofen 10 mg/d. However, this combination was of limited efficacy. This neurological consultation confirmed however the iatrogenic origin of dyskinesia. A treatment with clozapine is finally refused by the patient. By February 2014, an exertional dyspnea with a diaphragmatic spasm had appeared, followed by a permanent stridor due to a laryngospasm eight months later.

No further treatment was introduced. Alongside the neurological status, psychiatric condition deteriorated with worsening of depressive and anxiety symptoms, as well as suicidal thoughts. Tetrabenazine, clonazepam, and baclofen were stopped in November 2014 due to their lack of efficacy and the worsening of psychiatric symptoms. Anxiety and suicidal thoughts improved during a hospitalization when escitalopram was switched to mianserin 30 mg/d. MRI data from 2014 still showed no organic etiology to these symptoms.

The patient's neurological condition got increasingly worse with (i) orofacial dyskinesia—affecting facial muscles of the neck and diaphragmatic muscles—and (ii) a belly dancer's dyskinesia. Neurological deterioration rapidly led to a worsening of dyspnea (stage IV of NYHA [New York Heart Association] in November 2014) in a context of restrictive respiratory failure. Early 2015, several hospitalization cases had occurred in neurology and pulmonology clinics due to worsening of abnormal movements and respiratory failure. During these hospitalization cases, laryngospasm was improved by injections of botulinum toxin, but the beneficial effect fades very quickly. In March 2015, tetrabenazine 25 mg/d was finally reintroduced (with clonazepam 0.6 mg/d in June 2015) for dyskinesia to little efficacy. The patient's mental status remained stable until his death with mianserin. Mr. X reported at that time episodes of low mood and anxiety concomitant with dyspnea exacerbations. The patient died in July 2015 of a heart attack after exacerbation of dyspnea that lasted several days and malignant hyperthermia amid heat wave.

## 3. Discussion

Aripiprazole is reported to be a good clinical safety profile antipsychotic. However, caution is recommended and our patient had several tardive dyskinesia risk factors, including an advanced age, a diagnosis of mood disorder, and exposure to antipsychotic over a period longer than six months [6]. Overall, new data call into question the fact that tardive dyskinesia occurs less frequently with the APA compared to conventional neuroleptics [1, 7]. More specifically, one study suggests a ranking of the incidence of tardive dyskinesia associated with APA in which aripiprazole occupies a middle position: clozapine<quetiapine<aripiprazole<olanzapine=ziprasidone <risperidone [7].

Aripiprazole is associated with a better motor tolerance profile due to its specific dopaminergic action. Theoretical pharmacological data described that the blockage of more than 80% of D_2_ receptors led to a decrease of positive psychotic symptoms but also an increased risk of motor side effects. This might explain the fewer motor side effects associated with low doses of aripiprazole. Mamo et al. [8] have reported that 10 mg of aripiprazole resulted in more than 80% D_2_ striatal receptors occupancy (extrapyramidal side effects only observed in participants with occupancies exceeding 90%), whereas more recent studies have showed that (i) 5 mg of aripiprazole induced 55% of D_2_ striatal receptors occupancy and (ii) 6 mg of aripiprazole induced 74% striatal and 51% frontal D_2_ receptors occupancy [9]. Moreover, it is important to note that therapeutic low doses of aripiprazole (i.e., 2 and 5 mg) are associated with more extrastriatal than striatal occupancy [10]. Aripiprazole-induced tardive dyskinesia is reportedly rare in the literature. To our knowledge, only Peña et al. [11] reported a clinical case of tardive dyskinesia at low doses (5 mg/d). Like our case, their case was a middle-aged woman, of 60 years, who showed oral stereotypic and rapid dystonic movements after 4 months with aripiprazole. This data is partially consistent with our clinical case since both cases share risk factors, that is, advanced age and chronic use of aripiprazole. According to preclinical data, there are potential mechanisms besides the classical D_2_ receptors occupancy that might explain antipsychotic-induced motor dysfunctions. For example, Homer protein is implicated in numerous neurotransmitter regulations related to dopaminergic, glutamatergic, and GABAergic systems and the transgenic mice overexpressing the immediate early gene* Homer1a* in striatum expressed deterioration of motor abilities.* Homer1a* is known to be differentially induced by antipsychotics and gene expression appears to be induced (i) in the rat putamen by low acute doses of aripiprazole and in the cortex only with acute high doses and (ii) in the rat cortex and lateral striatum with chronic treatment [12]. This last condition can be related to the* Homer1a* striatal induction observed with haloperidol [12]. Thus, it is possible that antipsychotic-induced Homer family genes regulation played a role in striatal dysfunction and, in turn, in dyskinesia following chronic administration of aripiprazole.

Our case and its dramatic evolution, associated with other cases recently published (see, e.g., [11, 13–16]), incite reconsidering the real risk of tardive dyskinesia associated with aripiprazole. Finally, the use of aripiprazole for tardive dyskinesia improvement might not be a particularly safe care strategy given the specific risks of the molecule and the absence, so far, of long-term prospective studies [1].

## References

[1] Peña M. S., Yaltho T. C., Jankovic J. (2011). Tardive dyskinesia and other movement disorders secondary to aripiprazole. *Movement Disorders*.

[2] Duggal H. S. (2003). Aripiprazole-induced improvement in tardive dyskinesia. *Canadian Journal of Psychiatry*.

[3] Lykouras L., Rizos E., Gournellis R. (2007). Aripiprazole in the treatment of tardive dyskinesia induced by other atypical antipsychotics. *Progress in Neuro-Psychopharmacology & Biological Psychiatry*.

[4] Caykoylu A., Ekinci O., Yilmaz E. (2009). Resolution of risperidone-induced tardive dyskinesia with a switch to aripiprazole monotherapy. *Progress in Neuro-Psychopharmacology & Biological Psychiatry*.

[5] Schwartz T., Raza S. (2008). Aripiprazole (ability) and tardive dyskinesia. *Pharmacy and Therapeutics*.

[6] Dolder C. R., Jeste D. V. (2003). Incidence of tardive dyskinesia with typical versus atypical antipsychotics in very high risk patients. *Biological Psychiatry*.

[7] Woods S. W., Morgenstern H., Saksa J. R. (2010). Incidence of tardive dyskinesia with atypical versus conventional antipsychotic medications: A Prospective Cohort Study. *Journal of Clinical Psychiatry*.

[8] Mamo D., Graff A., Mizrahi R., Shammi C. M., Romeyer F., Kapur S. (2007). Differential effects of aripiprazole on D_2_, 5-HT_2_, and 5-HT_1A_ receptor occupancy in patients with schizophrenia: a triple tracer PET study. *American Journal of Psychiatry*.

[9] Murphy A., Dursun S., McKie S., Elliott R., Deakin J. F. W. (2016). An investigation into aripiprazole’s partial D2 agonist effects within the dorsolateral prefrontal cortex during working memory in healthy volunteers. *Psychopharmacology*.

[10] Kegeles L. S., Slifstein M., Frankle W. G. (2008). Dose-occupancy study of striatal and extrastriatal dopamine D2 receptors by aripiprazole in schizophrenia with PET and [18F] fallypride. *Neuropsychopharmacology*.

[11] Peña M. S., Yaltho T. C., Jankovic J. (2011). Tardive dyskinesia and other movement disorders secondary to aripiprazole. *Movement Disorders*.

[12] De Bartolomeis A., Tomasetti C., Iasevoli F. (2015). Update on the mechanism of action of aripiprazole: translational insights into antipsychotic strategies beyond dopamine receptor antagonism. *CNS Drugs*.

[13] Goyal R., Devi S. H. (2014). A case of aripiprazole induced tardive dyskinesia in a neuroleptic-naïve patient with two years of follow up. *Clinical Psychopharmacology and Neuroscience*.

[14] Sato K., Yoshida K., Higuchi H. (2012). Probable aripiprazole-induced tardive writer's cramp. *Journal of Neuropsychiatry and Clinical Neurosciences*.

[15] Alexander J., Bickerstaff S. (2013). Aripiprazole induced tardive dyskinesia—accruing evidence. *Australian and New Zealand Journal of Psychiatry*.

[16] Tomruk N. B., Saatcioglu O., Yildizhan E., Alpay N. (2011). Aripiprazole-induced tardive dyskinesia treated with quetiapine: a case report. *Acta Neuropsychiatrica*.

